# Individual and Contextual Variables as Predictors of MRI-Related Perceived Anxiety

**DOI:** 10.3390/bs13060458

**Published:** 2023-06-01

**Authors:** Margarida N. Farinha, Carla S. Semedo, António M. Diniz, Vasco Herédia

**Affiliations:** 1 Department of Psychology, School of Social Sciences, University of Évora, 7000-803 Évora, Portugal; 2 Research Centre in Education and Psychology (CIEP-UÉ), Department of Psychology, School of Social Sciences, University de Évora, 7000-803 Évora, Portugal; 3 Radiology Department, Hospital do Espírito Santo, EPE, Évora and Affidea-Évora, 9500-370 Évora, Portugal

**Keywords:** perceived anxiety, stress, education level, MRI, patient-centered service, radiology

## Abstract

Background: Magnetic resonance imaging (MRI) generates patient anxiety (PA) and, therefore, it is important to understand individual and contextual variables that may cause it. In study one, we explored those anxiety predictors. In study two, we examined the effect of the experience of MRI on PA comparing anxiety pre- to post-MRI. Methods: PA was measured with an anxiety and stress scale in an interview format. Data collection occurred at a public hospital with MRI outpatients aged 18 or older. In study one (*n* = 204), participants answered the questionnaire immediately after experiencing the MRI and the data were analyzed through structural equation modeling. In study two (*n* = 242), participants answered the questionnaire before and after the examination and the data were analyzed through Bayesian statistics. Results: Being female, having a higher education level (EL), and not receiving information about the examination predicts higher PA after MRI. Patients with prior information have a decrease in PA from pre- to post-MRI. Those who do not have no change in PA. In low-educated patients, PA also decreases and no changes occur in highly educated patients. Conclusion: This study provides health professionals with valuable indicators about patients who are more likely to perceive and express anxiety during MRI.

## 1. Introduction

Magnetic resonance imaging (MRI) is one of the most used diagnostic tools in radiology [1], since it is a non-invasive technique that uses strong magnetic fields with radio waves to generate images of the body physiological processes and its internal organs with high spatial and temporal resolution [2,3]. Although its biologically safe, non-invasive, and of a painless nature [2,4], the MRI procedure is frequently a source of fear and discomfort. Between 4 and 37% of patients experience anxiety due to this exam’s procedure [5,6], with 1 to 15% experiencing severe anxiety, claustrophobia, or panic attacks [5,7,8]; 10 to 14.5% of exams are prematurely interrupted [1,6]. These high values seem to be caused by physical properties of the scan such as the spatial constriction inside the scanner, the need to remain still, and the high level of noise produced [9,10]. In addition, other aspects such as the fear of experiencing pain, worry about the diagnosis, and perception of lack of control about the procedure [6,11,12] also contribute to patient anxiety during MRI.

Anxiety is defined as sense of uncontrollability towards any event perceived as dangerous or potentially negative to the person, who feels unable to control, predict, or obtain the desired outcome in a certain situation [13]. Anxiety can be experienced as an emotional state, being a subjective experience of the anticipation of possible negative outcomes [13], and as a physical state in which the body suffers physiological changes to prepare itself to respond to the perceived threat [14]. Thus, anxiety can be expressed through emotional symptoms such as fear or worry and through physical symptoms such as dry mouth, dizziness, and increased heart rate [15].

Perceived anxiety (PA) in MRI is known to change between the beginning and the end of the procedure. In some studies, PA is higher at the beginning of the exam than at the end of it [7,9,11,16], while, in others, no differences between PA pre- to post-MRI are found [1,17]. There seems to be a quadratic evolution in PA during the MRI scan [18] with it being higher at the beginning of the exam, reaching its peak when the patient is being inserted inside the scanner [8], and decreasing during the middle part of the exam, and, finally, increasing again at the end [18]. However, this last increase in PA at the end of the scan is rather difficult to understand because patients are told that the exam is coming to its end, which could influence that raise [18], possibly due to the patient’s urge of wanting to get out of the scanner. Nonetheless, the tendency seems to be that PA is greater at the beginning of the exam than during or after it, since the patients feel relieved and want to move on from the experience when it ends [19]. It is also worth noting that when PA is measured before the MRI, it seems to assess the level of anxiety the patient expects to feel during the exam, and serves the purpose of trying to predict the patients that are more prone to exhibit more anxiety symptoms during the scan (e.g., [11,20,21]), whereas when it is measured after the exam, it tends to be an indicator of the PA level the patient experienced during the scan (e.g., [7,17]).

## 2. Individual and Contextual Variables

What seems to cause anxiety in MRI can be divided in two groups of variables: (1) contextual, which includes aspects such as body part examined and what information is given to the patient; and (2) individual, with respect to unique characteristics of each patient such as gender and education level (EL).

Within contextual variables, differences in PA level can be found with respect to the body part examined in MRI. Exams of the head seem to be the ones that cause highest PA, leading to a greater need of sedatives for the patients to be able to undergo the procedure [22], and these are also the ones with higher interruptions rates [23]. Following head MRI, scans of the heart are also exams that cause high anxiety levels, since it is more demanding due to the need of some apnea moments [19]. In contrast, musculoskeletal MRI seems to be the exam that causes lower levels of anxiety [24,25,26], alongside with abdominal, pelvic, and breast MRI [27].

Offering information about the procedure is known to be effective in reducing patients PA during the scan [28]. Studies that compare patients that receive information prior to MRI against the ones who do not consistently show that the first group report less PA than the second (e.g., [7,12,14]). Moreover, the results of one intervention [7] show that the procedural information (i.e., information about how the exam is performed, including information about what the patient can expect and experience from the scan) is crucial to improve patients’ experience and reduce their PA. Lastly, information can be given not only through paper but also through video or audio [5,28] and it is also important to train health professionals in patient-centered communication [12,29]. The communication process between health professionals and their patients is particularly important because it can determine whether the patients will or will not adhere to the proposed treatment [30]. Therefore, health care professionals should improve their communication and interpersonal skills to provide better quality care to their patients [31]. However, although communication-based interventions that involve training staff’s communication skills improve patient adherence, anxiety, and satisfaction [12,32,33], the implementation of these skills tends to decrease over time [12], highlighting the significance of having on-going or periodic staff communication training [31].

Amongst individual variables, gender is the most commonly studied. Women tend to report higher PA than men in MRI [1,9,34] and also in other radiological procedures [24,25]. Women are also the group who express a greater need of sedatives to be able to undergo the MRI procedure [22]. Gender differences are more commonly found when PA is measured before the scan [8,20,35], although there is one study [15] in which women reported higher PA than men both before and after MRI. However, one can also find studies that failed to find gender differences in MRI anxiety [11,27]. Additionally, we might think, similar to other authors [24,25], that age could be a PA predictor, but results from these studies show that there are no significant differences in PA levels. We could not find studies that included an analysis of the relationship between PA in MRI and patients’ EL. Despite that, this relationship was studied in other radiological procedures, which included MRI, and in which the data was collected before the procedure [24]. Results show that people with “higher education” report less PA than the ones with lower education, “high school”, “middle school”, and “elementary or below” [36]. The authors suggest that these results are due to the higher level of patient’s education, enhancing the acquisition of information about the exam, the disease, and its treatment.

## 3. The Current Study

MRI-related anxiety is an increasing problem, and, therefore, it is essential to better understand what variables cause patient’s anxiety so that measures can be taken to reduce it. Most studies about anxiety in MRI tend to focus on group differences (e.g., [8,11,17]), with fewer investigations (e.g., [20,21]) exploring what are the predictors of anxiety in MRI. Moreover, studies show that PA differs between the beginning and the end of the examination, but the results can be contradictory (e.g., [9,11,24]) and there is a need to clarify what going through the experience of MRI does to patients’ PA. Thus, the current investigation is composed of two sequential studies with different objectives. With the first study, we aimed to explore the variables that can predict anxiety in MRI, and with the second study, we intended to understand what effect (increase, decrease, or none) the actual experience of MRI has on patient’s PA, by comparing the levels of anxiety of pre- and post-MRI.

## 4. Study One

The contextual predictors in study are the body part examined in MRI and the procedural information given by the physician prescribing the exam (henceforth throughout information from the prescriber). The first was already studied as a predictor of anxiety, but in different radiological procedures and not in MRI alone (e.g., [24]) and the only study found that tried to identify the predictors of panic symptoms in MRI (e.g., [20]) did not include body part examined. As for the latter, giving information about the exam to the patient has been studied to reduce anxiety (e.g., [37,38]), and not as a regression predictor.

Lastly, the individual variables in study are gender and patients’ EL. Gender is a variable included in many studies (e.g., [9,24]), but it shows contradictory results, since there are studies where gender differences are found [8,15], and other where they do not exist [11,27]. As for the EL, to the best of our knowledge, its relationship with MRI anxiety has not been studied yet, only with regards to different radiological procedures [36].

The prediction relationships were analyzed through structural equation modeling, with PA being the latent variable in a multiple regression model, also known as a multiple indicators and multiple causes (MIMIC) [39] model. We expect the four variables under investigation to be predictors of PA, with females, less-educated people, patients with no information from the prescriber, and head MRI presenting higher PA levels.

### 4.1. Materials and Methods

#### 4.1.1. Participants

A sample of 204 participants (age range = 18–66 years; median = 57) was collected at a public hospital. Participants were mostly female (65.69%), and most participants (68.14%) reported receiving no information from the prescriber and were undergoing MRI for the first time (58.8%). As shown in Table 1, participants were more than 44 years old (77.45%) and had completed at least five years of education (58.82%), and head MRI was the most common exam (31.37%).

Taking into account the possible influence of hormonal fluctuations due to different phases of the reproductive lifespan on the PA levels of women, we tested if there were differences in women’s PA levels. Since menopause typically occurs between 45 and 55 years [40], we divided women into three age groups: before menopause (less than 45 years), during menopause (between 45 and 55 years), and after menopause (above 55 years). Through ANOVA, we find no statistically significant differences between groups (F(2, 131) = 2.066, *p* = 0.131), which indicates that, in this study, there is no influence of the phase of the reproductive lifespan on women’s PA levels.

#### 4.1.2. Materials

Characterization questionnaire. A short questionnaire was used to collect demographic data, namely, age, gender, and EL, and information about the exam’s contextual aspects, namely, body part examined and if the patient received information from the prescriber.

Depression, anxiety, and stress scales (DASS). Anxiety and stress scales of the Depression, anxiety, and stress scales (DASS) [41] were used to measure PA. Each factor is composed of seven items, rated on a four-point Likert-type scale, ranging from “did not apply to me at all” to “applied to me very much”. Anxiety subscale items translate fear-related symptoms such as “I felt scared without any good reason”, and stress subscale items imply perceived states of persistent arousal and tension such as “I found it difficult to relax”.

The DASS was translated into the Portuguese language and validated [42]. Semedo et al. [12] validated the Portuguese version in a sample similar to the one used in the present study, since it was a very different population from the university students sample used in the Portuguese validation [42]. This validation was made with data collected prior to the MRI [12]. The authors performed the structural validation of anxiety and stress subscales through a confirmatory factor analysis and obtained a high correlation (0.95) between both factors. Likewise, the 14 items were collapsed into a single factor—PA.

#### 4.1.3. Procedure

Data collection. Data collection occurred before the SARS-CoV-2 pandemic (July 2014–January 2017) according to the guidelines outlined in the Declaration of Helsinki, after the approval of the institution and the Ethics Committee for Research in the Areas of Human Health and Wellbeing of the University of Évora, inviting eligible patients to participate in the study. The preconditions were: MRI outpatients aged 18 or older without any medical condition that could affect MRI experience (e.g., anxiety disorders). Participants informed consent was obtained prior to study commencement. They answered the questionnaire immediately after experiencing the MRI in a suite with a magnet room operating a 1.5 T SIGNATM MRI (GE Healthcare, Chicago, IL, USA). The questionnaire was applied in another room with an interview format by trained psychology master’s students, so that illiterate participants would be included in the study, since this is a commonly overlooked population (e.g., [9,24]).

Statistical analysis. IBM SPSS Statistics for Windows (version 24) was used to perform data descriptive analysis. Prior to the MIMIC model estimation, structural validation of DASS anxiety and stress subscales (henceforth throughout, PA) was assessed through confirmatory factor analysis with LISREL 8.80, using the underlying bivariate normal approach [43] because DASS items are ordinal [42]. Likewise, based on polychoric correlations and respective asymptotic covariances, the Satorra–Bentler scaled correction for non-normality produces robust maximum likelihood estimates and chi-squares (SBχ^2^) were used [44]. Model fit was established by examining attending benchmarks [45] values for the comparative fit index (CFI; should be close to or above 0.95), the root-mean-square error of approximation (RMSEA; values close to or below 0.06), and the standardized root-mean-square residual (SRMR; values close or below 0.80).

Convergent validity (CV) and composite reliability (CR) were also examined [46]. The first is based on items’ average variance extracted by respective factor (AVE) and should be at least 0.50; the latter is recommended to be of 0.80 for group comparisons [47].

The predictive relationships within the MIMIC model were examined according to Cohen’s effect sizes benchmarks (*R*^2^ = 0.02, small; *R*^2^ = 0.13, medium; *R*^2^ = 0.26, large) [48].

### 4.2. Results

The PA model shows an excellent fit to the empirical data (SBχ^2^ = 85.264, *df* = 77; RMSEA [IC 90%] = 0.023 [0.000, 0.047]; SRMR = 0.036; CFI = 0.999). The model’s psychometric properties (AVE and CR), which are excellent for use in the MIMIC model test, are presented in Table 2.

**Figure 1:**
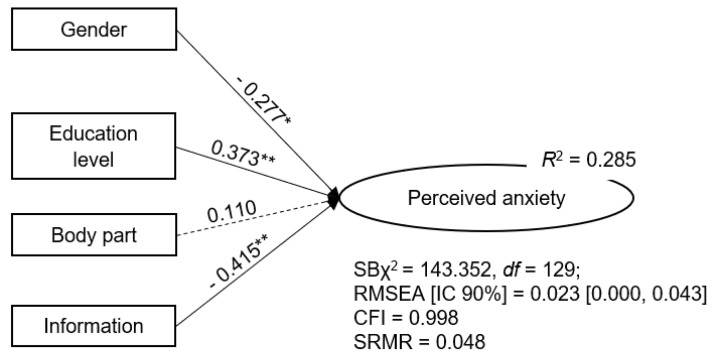
Predictive model of perceived anxiety in magnetic resonance imaging: results. **Notes**: Standardized solution. Gender: 1 = female, 2 = male. Education: 1 = elementary school or below, 2 = middle school or above. Body part examined: 1 = head, 2 = musculoskeletal, 3 = abdominal, 4 = pelvic, 5 = breast. Information about the procedure given by the physician that prescribed the exam: 1 = received, 2 = did not receive. Dashed arrows = non-significant relationships. *R*^2^ (multiple regression determination coefficient) = amount of variance captured by the predictors. Standardized regression coefficients (β) values above the arrows. * *p* < 0.05. ** *p* < 0.01.

The tested MIMIC model (Figure 1) is very well fitted to the data, with gender, EL, and the information from the prescriber being predictors of PA in MRI, with a large effect size (*R*^2^). Three of the four tested predictors contribute to this large effect size (gender, EL, and information from the prescriber), with information from the prescriber and EL being the most important factors, since they are significant for *p* < 0.01.

### 4.3. Discussion

The objective of study one was to explore the predictive influence of having received information from the prescriber about the procedure and body part examined (contextual variables), as well as gender and EL (individual variables) on PA in MRI. We expected that being female (e.g., [34]), having lower education (e.g., [36]), and not receiving information about the procedure (e.g., [49]) would be predictors of PA. As for the body part examined, we expected the head MRI to be the best predictor of PA. The MIMIC model shows that 28.5% of PA is predicted by gender, EL, and information from the prescriber. Body part examined is, against expectations (e.g., [22,26]), not a predictor of PA.

The results about gender are similar to previous studies where women report higher PA than men in radiology procedures [24] and in studies with MRI-specific procedures [8,9,15]. Gender differences in expression of PA, fear, or stress are common in different contexts, as well as across the lifespan, with girls and women reporting higher levels of PA than boys and men [50]. However, it might be possible that this gender differences do not represent a real experience of higher PA from women. This might be due to women having more openness to express negative feelings such as anxiety, fear, stress, and sadness because they are encouraged to do so since childhood, whereas boys tend to be punished or criticized [51]. Thus, it is possible that the gender differences found are more an expression of social desirability than a real or accurate higher susceptibility from women to experience more anxiety. This idea can be supported by studies where no gender differences in PA in MRI were found [11,27].

As for information given to the patient, the present results align with other investigations that highlight the efficacy in patients PA reduction through information offering [7,49], especially procedural information [14,37]. Patients state that communication with health professionals is the most important factor to promote their comfort [52], and want to receive information before the procedure, as well as have the opportunity to ask questions. Hence, health care professionals responsible for carrying out the MRI examinations (e.g., physicians, nurses, radiology technicians) have a crucial role in mitigating patient’s PA [33,53]. Staff must develop relational competences that allows them to provide a patient-centered service (e.g., [12,54]) that will, in turn, improve patients’ anxiety and satisfaction [28].

The only study found that examined the relationship between patients’ EL and PA [36] showed different results than those we report in the current study, since we found out that having more education predicts higher levels of PA. However, three methodological differences in our study may have play a role in the results we found: the variable EL was operationalized in two levels (elementary school or below; middle school or above) instead of four; the instrument we used to measure perceived anxiety, has items more specific for the radiologic procedures domain than the state–trait anxiety inventory used by others [36]; and the data collection was performed after MRI instead of before. Moreover, less-educated people tend to have less health literacy [55], that is, less capability to search, understand, and use information to make health-related decisions [56]. These people seem to have more trust in health care professionals than the ones with a higher education level due to being more dependent on the information they obtain from these professionals [57]. Thus, the lower levels of PA in less-educated patients might be explained through the trust they have in health professionals (e.g., “doctors know about health and what I need to do”), which can make them feel that they do not need any explanations about medical procedures. On the contrary, more-educated people, having higher competencies in autonomous health information searches, might be less dependent on health professionals’ information, which may be anxiogenic because they could be preparing themselves for the MRI with unnecessary or irrelevant information [58].

Body part examined is not a good predictor of PA, even though some studies report different levels of PA in examinations of different body parts (e.g., [19,21,34]). It is worth noting that studies where these differences are found tend to compare PA levels measured before the MRI (e.g., [21,24,25]) and not after, as in our study. Therefore, this discrepancy of results between the current and previous investigations can indicate that patients anticipate more anxiety before MRI than the examination makes them effectively feel post-MRI. Alongside this, the lack of statistical significance in the present study for body part examined might mean that this is not a particularly important feature of MRI examinations and that other aspects such as information given to the patients, their gender, and education level are more important.

## 5. Study Two

The objective of study two was to compare patients’ PA before and after the MRI examination with the predictive variables of study one (i.e., with *p* < 0.01). Thus, we intend to compare the levels of anxiety that patients expect to feel in MRI (PA measured before the exam) with the anxiety they experienced during the examination (PA measured after MRI). The hypothesis that sustains this goal derives from the different results found in previous studies, some revealing higher anxiety before MRI [11], others showing no relation between anxiety and the time data were collected [1,17]. Moreover, we examined this hypothesis accordingly to differences in patient’s EL and in procedural information (i.e., if the patients received information from the prescriber).

### 5.1. Materials and Methods

#### 5.1.1. Participants

A sample of 242 participants (age range = 18–88 years; median = 57) was obtained, measuring PA both before and after the examination. The majority of participants were from study one (*n* = 204), were mostly female (62.81%), and most participants (66.53%) reported receiving no information from the prescriber and were undergoing MRI for the first time (56.8%). As Table 3 shows, most participants are more than 44 years of age (77.28%) and have completed at least five years of education (56.20%).

Similarly to study one, we find no statistically significant differences between women before, during, and after menopause for both PA before (F(2, 149) = 2.357, *p* = 0.098) and after (F(2, 149) = 2.193, *p* = 0.115) the MRI.

#### 5.1.2. Materials

The same as study one.

#### 5.1.3. Procedure

Data collection. Data were collected in an identical way to study one, except that in study two, participants were interviewed between February 2018 and September 2019, both before and after the MRI. As in study one, data collection was carried out according to the guidelines outlined in the Declaration of Helsinki, after the approval of the institution and the Ethics Committee for Research in the Areas of Human Health and Wellbeing of the University of Évora. Participants informed consent was collected prior to study commencement.

Statistical analysis. Open-source statistical package JASP (JASP Team, 2021, Version 0.16, https://jasp-stats.org/ accessed on 1 December 2021) was used to perform Bayesian data analysis. A Bayesian mixed ANOVA was performed to evaluate if there were any changes in PA due to the experience of MRI in a two (pre- and post-PA) within-subjects x two (EL) × two (information from the prescriber) between-subjects (see Table 4) factorial design. This approach allows the examination of the measured factor (PA) against two contending hypotheses (H1 vs. H0) with 50% prior odds each, testing the strength of evidence without threats to the reliability of effect size analysis originating through classic inference [59]. A Bayes factor (BF10) equal to five per example means that the data are five times more likely to occur under H1 than H0. Results were analyzed following JASP’s criteria [60]: effects higher than 100 represent extreme evidence for H1; between 30–100, very strong evidence for H1; between 10–30, strong evidence for H1; between 3–10, moderate evidence for H1; between 1–3, anecdotal evidence for H1; 1, no evidence. Baws factors [61] were computed for each main effect and interaction, resulting in a BF representing the evidence weight of models containing the effect versus the evidence of equivalent models stripped of that effect. Decomposed interactions were tested through repeated measures ANOVA and, in these cases, BFs represents the evidence weight of models containing the effect against all models without that effect.

### 5.2. Results

Table 4 shows the results of the analysis of the effects of study variables and their interactions. There is extreme evidence for H1 in the interactions between PA × EL and PA × information from the prescriber.

Table 5 presents the results of the decomposition of the interactions with Bayesian repeated-measure ANOVAs for PA in each level of the study variables, with PA level means for both pre- and post-MRI. As we can see, there is very strong evidence for H1 for patients that receive information from the prescriber prior to MRI, meaning that there is a variation in patients’ PA level pre- and post-MRI. Likewise, very strong evidence for H1 is also found for patients with low or no education.

Overall, these results show that patients with lower education and patients who receive information from the prescriber show more anxiety than what the exam seems to cause to them, and that undergoing the procedure decreases that anxiety. Moreover, as we can see in Table 5, there is an inversion pattern on the means of PA pre- and post-MRI between the levels of the examined variables: EL and information from the prescriber. As for people with no prior information or with higher EL, there is no effect on PA due to going through the MRI experience.

### 5.3. Discussion

With study two, we aimed to further explore the variables we found to be the most important predictors of PA in study one (EL and information from the prescriber), comparing patients’ PA before and after the MRI to understand what effect the experience of MRI has on patients’ PA.

Patients that receive information from the prescriber prior to MRI report higher PA before the examination than those who do not have information. However, those who receive information have a significant decrease in PA from pre- to post-MRI and patients with no prior information have no change in PA levels. The interpretation of these results is related to the study one findings. When giving information about medical procedures to patients, it is important to share relevant and sufficient information to prepare them for the procedure [58], and that information should be tailored to their needs. The higher levels of PA in patients with prior information from the prescriber, compared to those that do not receive it, may represent an under- or oversharing of information. In the first case, it is possible that patients with little information about the examination have some doubts or questions about the procedure caused by the limited information received. On the other hand, if a patient receives too much information, they could have had difficulties in understanding it all, generating confusion and more anxiety towards the examination. Also, although patients have received information from the prescriber, we do not control for if they have obtained information about MRI from other sources (e.g., family, friends, TV, internet). If so, having information from non-expert sources could result in misconceptions of MRI [62] and increased pre-MRI anxiety. Nonetheless, having information prior to MRI results in a significant decrease in PA from pre- to post-examination. With prior information, patients know what to expect and even if they feel fear or anxiety at first, the coherence between the information given and the experience in the scanner seems to alleviate that anxious state.

The same pattern is found in patients with low EL: these patients report higher pre-MRI anxiety than those with a higher EL, but only the less-educated patients have a variation in their PA levels pre- and post-MRI (i.e., their PA decreases). More-educated patients tend to have more knowledge about the MRI procedure [62] and more health literacy [63]. However, the results of the present study seem to indicate that having more information and/or capacity to interpret that information can be a set back and produce more PA [58]. Also, when highly anxious in MRI, the anxiety may persist or even increase [64]. Hence, it seems that more-educated people tend to overthink the received information during the examination, probably trying to link what is happening to what it was said about what would happen, resulting in a constant level of PA. On the other hand, less-educated patients might feel that the information received is enough and corresponds to the experience of MRI, resulting in a significant decrease in PA from pre- to post-MRI.

## 6. General Discussion

Taking together the results of both studies, we can reinforce the idea that training health care professionals and giving information to patients is essential to manage patients PA, even though in some cases (i.e., more-educated patients) it can be prejudicial [58]. Despite its benefits, interventions aiming to improve the skills of health professionals entail, although reduced, some unwanted costs for health care services [28]. It should also be noted that, sometimes, the physician prescribing the MRI does not know the examination procedure well enough, as experienced by radiologists [65]. In that situation, prescribers should receive some training to better understand the MRI procedure and be able to give patients information that allows them to know what to expect from the examination and be prepared for the procedure.

Considering the importance of providing information for patients’ well-being and for the cost reduction involved, and given the relationship between PA and the duration, interruption, and need to repeat the scan [2,10], it is crucial to invest, in some way, in this practice. Information can be provided using a phone call or video [38], as well as through a prior appointment at the MRI location to explain the examination procedure to the patient and/or show the scanner room [28]. This information can also be offered through, for example, booklets with detailed information about the procedure and what the user can expect from the experience [7], or with images illustrating the procedure and simple relaxation strategies [37]. In addition, if it is not possible to provide information to the patient in advance of the exam, this can be transmitted moments before the exam by radiology technicians (e.g., [14]), since this practice also results in lower levels of user’s PA.

It is also relevant to understand what type of information patients want and how they prefer to obtain it. For example, in a qualitative study [66] participants indicated the need to receive, along with written information, visual information. As they report, it is flashier, and they have more attention when there are images. In the same study, participants mention that obtaining information through a DVD helps prepare for the MR experience, knowing exactly what they can expect to happen. Thus, in the case of women and people with a higher EL (i.e., those who report higher levels of AP), it may be appropriate to carry out a prior appointment to clarify doubts about the procedure, show the exam room to the user to create a familiarization with the physical environment, and or provide a video that exemplifies the MRI procedure. It is also important that information given through video, or photos, to familiarize patients with MRI and allow them to obtain the needed information, is friendly for patients without reading competencies. If it is impossible to provide information to users before the exam, the staff can also improve patients’ experience during the exam by maintaining verbal contact with them through the intercom [67]. Hence, the technicians carrying out the exam can explain to the patient what is happening, what is next, how much time is left, and if the exam is proceeding as expected. The results presented here show that PA is similar between groups before MRI, but after the examination there are some differences. This study shows that having a higher EL and less information predicts higher PA in MRI (study one), and that PA only decreases from pre- to post-MRI for patients with a lower EL and that have prior information about the procedure (study two). It is interesting to focus on EL, which, as far as we know, is the novelty study variable to the MRI scientific literature (the only study we found included a broader set of radiological exams [36]). When we look at the effect of going through the scanner, we see that only the less-educated people have a significant reduction in PA. This can mean the overall experience of MRI is more anxiogenic to patients with more education, since only the ones with a lower EL have a decrease in PA. Therefore, more-educated patients seem to need tailored patient-centered services, attending to their concerns and stressors during MRI. It is important to note that the presented results do not imply a greater need of attention in MRI from more-educated people. This study shows that healthcare professionals must be able to implement patient-centered communication strategies [68], seeking to establish a relationship of trust with them, recognizing their state of anxiety, validating that state, and trying to relax them (e.g., saying that what the patient is experiencing is frequent and that they should, for example, try to breathe deeply and slowly). After the exam, patients may continue to feel and express anxiety [18], and, therefore, the radiology technician(s) should pay attention to the patient’s condition, prepare to answer any questions, and provide enough time for them to relax before leaving. Besides EL and information from the prescriber, gender (i.e., being female) is also a predictor of increased PA in MRI, as study one results show. However, results about gender are not entirely consistent across the literature [9,11,27,34] and it is significant only for *p* < 0.05. Given that, and the concerns expressed in study one discussion related to a possible artificiality of this results based on social desirability, in order not to incur in *p*-hacking [69] gender was not considered as an interest variable in study two.

## 7. Limitations, Strengths, and Further Studies

One of the limitations of this study is that it is not possible to infer causal relationships because of its cross-sectional design, and there are some difficulties in generalizing results due to the non-probabilistic sampling [70]. Regarding the chosen instrument, it is known that self-report instruments are prone to social desirability bias [71,72]. Administering it through interview may have exacerbated that bias, with patients distorting their responses to describe themselves as less anxious than they felt, as noted by the interviewers during the data collection procedure. However, this was a considered and necessary setback, so that people with no education could be included in the study [73]. Some notes can also be made regarding the operationalization of body part examined. This variable was considered based on the body region examined in MRI, but we did not control the reason/clinical condition that led to the examination or the position the patient remained in during it. For example, breast MRI tends to be related to screening or to a previous diagnosis of breast cancer [74] and that being so, the reason for performing the examination and concerns about its results [6] might exacerbate PA and even help to understand gender differences. As for the position of the patient during the examination (prone vs. supine), this aspect may have affected the results since the position depends on the body part examined [27] and influences patients’ PA [75]. Also, MRI examination can be performed with or without intravenous contrast and this is another uncontrolled aspect of the procedure that may have affected our findings in an unknown way. Lastly, other variables such as radiologists’ different communication skills can jeopardize the standardized information about the exam [12] and, therefore, could also be included as covariates in the study. However, if those covariates were considered, the model complexity would threaten parsimony.

Despite its limitations, the current study also has its strengths. One of the major strengths and new contributions of the current study is the inclusion of patients with no education (i.e., without reading or writing competences). Most studies tend to use alphabetization as an inclusion criterion (e.g., [9,24,64]), resulting in sub-representation of this group in a clinical context. However, these people also benefit from clinical health care services and their experiences should also be considered to better understand patient’s needs and provide better services. Concerning the statistical techniques applied, it is relevant to note that the effect sizes obtained, according to Cohen’s [48] benchmarks, are greater than the usually reported in social sciences studies (i.e., weak effect sizes [76]).

This investigation is also a replication of Semedo et al. [12], validating the instrument in post-MRI administration since, in that study, PA is validated prior to MRI. Having obtained good psychometric properties, this study strengthens the chosen instrument, which is different from most of the literature reviewed that uses STAI (e.g., [6,11,14,36,38]). Given the difference between the operationalization of PA across studies, it is understandable that the results may differ, as it is the case of EL, in which the results found in our study are opposite to Re et al. [36] that use STAI to measure PA.

Further studies must replicate the present study in independent samples to assess the generalizability of the prediction model studied [77], with preference for probabilistic sampling or, if impossible, with a greater sample. It might also be worthy to include some unconsidered variables such as how many previous MRIs the patient has experienced, the quality of those experiences [26], and the respective PA, and the source of information chosen to seek information from about the exam. Other individual variables such as patient’s clinical condition or reason for the examination, and procedural variables such as position during MRI, method of entrance in the scanner, or intravenous contrast administration should be considered in further studies. Furthermore, given the specificity of breast MRI (i.e., performed with intravenous contrast that may exacerbate PA, but in prone position that can mitigate PA) it might be useful to study this variable singly or not to consider it if body part examined is a study variable.

As for EL, since it is a recurrently unconsidered variable, and in the current study it is dichotomized, it could be useful to operationalize it in more levels (e.g., no education, basic education, middle school, high school, higher education) to better understand the group that is more prone to experience anxiety in MRI and benefit more from a health care service tailored to their needs [33]. Lastly, social desirability implicated in self-report instruments can be reduced, without needing to exclude unalphabetized patients, using objective measures of PA [8,18], such as blood oxygen saturation [1], levels of cortisol and prolactin [14], and respiratory rate [6]. In addition, combining objective and subjective measures of PA can help clarify and better understand the artificiality of gender differences suggested here. In addition, objective measures enable the direct assessment of PA during MRI, without the need for the patient to report PA beforehand (e.g., [11]) or retrospectively (e.g., [7]).

## 8. Conclusions

The current study helps strengthen the idea that providing information about the procedure to the patient is one of the main ways to prevent and/or reduce PA in MRI, since this is its most important predictor. Alongside procedural information, EL also emerges as an important predictor of MRI anxiety, which helps to understand PA causes and adds to the scientific literature by exploring and acknowledging a frequently overlooked variable in the MRI context. Patients with low or no literacy competencies tend to be excluded from studies because this aspect demands more training/skills, attention, and time consumption from the person who administer the instruments [70].

The results of the current investigation are important for health care professionals, especially in radiology, since they help them identify the patients that are more prone to exhibit anxiety in MRI (women, patients with a higher EL, and without prior information from the prescriber) so that they can give a personalized service tailored to patients’ needs (e.g., give information, answer questions). At a local level, the reported results allow the radiology department where data were collected to become aware of their current patient care practices and identify ways to improve their service and, therefore, patient’s experience.

In conclusion, the results of the present study contribute to a better understanding of the predictors of PA in MRI (being female, having a higher EL, and not receiving information from the prescriber), giving health professionals valuable indicators about patients who are more likely to perceive and express anxiety in MR, and those who are most likely to benefit from specific strategies implemented by the staff to mitigate it.

## Figures and Tables

**Table 1:** Participants characteristics and MRI contextual aspects.

	*n*	%
Age		
18–44	46	22.55
45–64	98	48.04
≥65	60	29.41
Education level		
Low (up to 4 years completed) ^a^	84	41.18
No education	8	3.92
High (at least 5 years completed)	120	58.82
Body part examined		
Head	64	31.37
Musculoskeletal	36	17.64
Abdominal	37	18.14
Pelvic	37	18.14
Breast	30	14.71

^a^ Including participants with no education.

**Table 2:** PA model: robust maximum likelihood estimates, average variance extracted, and composite reliability.

Item	β	*R* ^2^
1. I found it hard to wind down	0.947	0.897
2. I was aware of dryness of my mouth	0.659	0.434
3. I experienced breathing difficulty	0.810	0.656
4. I tended to over-react to situations	0.905	0.819
5. I experienced trembling (e.g., in the hands)	0.794	0.630
6. I felt that I was using a lot of nervous energy	0.908	0.824
7. I was worried about situations in which I might panic and make a fool of myself	0.861	0.741
8. I found myself getting agitated	0.930	0.865
9. I found it difficult to relax	0.942	0.887
10. I was intolerant of anything that kept me from getting on with what I was doing	0.881	0.776
11. I felt I was close to panic	0.951	0.904
12. I felt that I was rather touchy	0.868	0.753
13. I was aware of the action of my heart in the absence of physical exertion	0.824	0.679
14. I felt scared without any good reason	0.970	0.941
AVE	0.882	
CR	0.990	

**Notes:** β = completely standardized factor loading; *R*^2^ (communality) = 1 − ε (standardized residual); AVE = average variance extracted; CR = composite reliability.

**Table 3:** Participants characteristics.

	*n*	%
Age		
18–44	55	22.72
45–64	117	48.35
≥65	70	28.93
Education level		
Low (up to 4 years completed) ^a^	106	43.80
No education	8	3.31
High (at least 5 years completed)	136	56.20

^a^ Including participants with no education.

**Table 4:** Bayesian mixed ANOVA’s results.

Factors	P_(incl)_	P_(excl)_	P_(incl|data)_	P_(excl|data)_	BF_10_
PA	0.263	0.263	0.001	0.003	0.347
Education level	0.263	0.263	7.070 × 10^−4^	0.005	0.151
PA × education level	0.263	0.263	0.912	4.507 × 10^−4^	**2022.938**
Information	0.263	0.263	0.004	0.025	0.170
PA × information	0.263	0.263	0.886	0.007	**135.718**
Education level × information	0.263	0.263	0.383	0.508	0.755
PA × education level × information	0.053	0.053	0.083	0.380	0.217

**Notes:** Information = procedural information given by the physician prescribing the exam. P_(incl)_ = prior inclusion probability; P_(excl)_ = prior exclusion probability; P_(incl|data)_ = posterior inclusion probability; P_(excl|data)_ = posterior exclusion probability; BF_10_ = effect size. Extreme effect sizes in bold.

**Table 5:** Bayesian repeated-measures ANOVA results.

Factors	*M* of PA		Analysis of Effects		
	Pre-MRI	Post-MRI	P_(incl)_	P_(incl|data)_	BF_10_
Received information	6.84	3.30	0.50	0.99	**81.81**
Did not receive information	5.24	5.53	0.5	0.12	0.13
Low education	6.95	3.29	0.50	0.99	**88.50**
High education	4.86	5.94	0.50	0.26	0.35

**Notes**: Model comparison = all models include subject. P_(incl)_ = prior inclusion probability; P_(incl|data)_ = posterior inclusion probability; BF10 = effect size. Very strong effect sizes in bold.

## Data Availability

The data presented in this study are partially available on request from the corresponding author. The data are not publicly available due to privacy of participants, who agreed to share personal information only with the investigation team.

## References

[1] Arda K.N., Akay S., Yetkin S. (2020). Is there a relationship between oxygen saturation and MRI-induced anxiety? A prospective study. Clin. Imaging.

[2] Almutlaq Z.M. (2018). Discussion of the causes, effect and potential methods of alleviating patient anxiety when undergoing magnetic resonance imaging (MRI). Egypt. J. Hosp. Med..

[3] Siafaka P.I., Okur N.Ü., Karantas I.D., Okur M.E., Gündoğdu E.A. (2021). Current update on nanoplatforms as therapeutic and diagnostic tools: A review for the materials used as nanotheranostics and imaging modalities. Asian J. Pharm. Sci..

[4] Nakarada-Kordic I., Reay S., Bennett G., Kruse J., Lydon A.M., Sim J. (2020). Can virtual reality simulation prepare patients for an MRI experience?. Radiography.

[5] Cable J., Bormann S. (2015). Anxiety during “first time” magnetic resonance imaging. J. Health Res..

[6] Dziuda Ł., Zieliński P., Baran P., Krej M., Kopka L. (2019). A study of the relationship between the level of anxiety declared by MRI patients in the STAI questionnaire and their respiratory rate acquired by a fibre-optic sensor system. Sci. Rep..

[7] Bolejko A., Hagell P. (2021). Effects of an information booklet on patient anxiety and satisfaction with information in magnetic resonance imaging: A randomized, single-blind, placebo-controlled trial. Radiography.

[8] van Minde D., Klaming L., Weda H. (2014). Pinpointing moments of high anxiety during an MRI examination. Int. J. Behav. Med..

[9] Ahlander B.M., Engvall J., Ericsson E. (2020). Anxiety during magnetic resonance imaging of the spine in relation to scanner design and size. Radiography.

[10] Nguyen X.V., Tahir S., Bresnahan B.W., Andre J.B., Lang E.V., Mossa-Basha M., Mayr N.A., Bourekas E.C. (2020). Prevalence and Financial Impact of Claustrophobia, Anxiety, Patient Motion, and Other Patient Events in Magnetic Resonance Imaging. Top. Magn. Reason. Imaging.

[11] Klaming L., van Minde D., Weda H., Nielsen T., Duijm L.E. (2015). The Relation Between Anticipatory Anxiety and Movement During an MR Examination. Acad. Radiol..

[12] Semedo C.S., Diniz A.M., Herédia V. (2020). Training health professionals in patient-centered communication during magnetic resonance imaging to reduce patients’ perceived anxiety. Patient Educ. Couns..

[13] Barlow D.H. (2000). Unraveling the mysteries of anxiety and its disorders from the perspective of emotion theory. Am. Psychol..

[14] Tazegul G., Etcioglu E., Yildiz F., Yildiz R., Tuney D. (2015). Can MRI related patient anxiety be prevented?. Magn. Reason. Imaging.

[15] Thu H., Etcioglu E., Yildiz F., Tuney D. (2015). Factors associated with increased anxiety in the MRI waiting room. J. Radiol. Nurs..

[16] Katz R.C., Wilson L., Frazer N. (1994). Anxiety and its determinants in patients undergoing magnetic resonance imaging. J. Behav. Ther. Exp. Psychiatry.

[17] Baran P., Truszczynski O., Dziuda L. (2015). Anxiety in patients undergoing magnetic resonance imaging. Pol. J. Aviat. Med. Psychol..

[18] Chapman H.A., Bernier D., Rusak B. (2010). MRI-related anxiety levels change within and between repeated scanning sessions. Psychiatry Res..

[19] Ahlander B.M., Årestedt K., Engvall J., Maret E., Ericsson E. (2016). Development and validation of a questionnaire evaluating patient anxiety during Magnetic Resonance Imaging: The Magnetic Resonance Imaging-Anxiety Questionnaire (MRI-AQ). J. Adv. Nurs..

[20] Harris L.M., Menzies R.G., Robinson J. (2001). Predictors of panic symptoms during magnetic resonance imaging scans. Int. J. Behav. Med..

[21] Napp A.E., Enders J., Roehle R., Diederichs G., Rief M., Zimmermann E., Martus P., Dewey M. (2017). Analysis and Prediction of Claustrophobia during MR Imaging with the Claustrophobia Questionnaire: An Observational Prospective 18-month Single-Center Study of 6500 Patients. Radiology.

[22] Murphy K.J., Brunberg J.A. (1997). Adult claustrophobia, anxiety and sedation in MRI. Magn. Reason. Imaging.

[23] Norbash A., Yucel K., Yuh W., Doros G., Ajam A., Lang E., Pauker S., Mayr N. (2016). Effect of team training on improving MRI study completion rates and no-show rates. J. Magn. Reason. Imaging.

[24] Forshaw K.L., Boyes A.W., Carey M.L., Hall A.E., Symonds M., Brown S., Sanson-Fisher R.W. (2018). Raised Anxiety Levels Among Outpatients Preparing to Undergo a Medical Imaging Procedure: Prevalence and Correlates. J. Am. Coll. Radiol..

[25] Heyer C.M., Thüring J., Lemburg S.P., Kreddig N., Hasenbring M., Dohna M., Nicolas V. (2015). Anxiety of patients undergoing CT imaging-an underestimated problem?. Acad. Radiol..

[26] MacKenzie R., Sims C., Owens R.G., Dixon A.K. (1995). Patients’ perceptions of magnetic resonance imaging. Clin. Radiol..

[27] Eshed I., Althoff C.E., Hamm B., Hermann K.G. (2007). Claustrophobia and premature termination of magnetic resonance imaging examinations. J. Magn. Reason. Imaging.

[28] Ajam A.A., Tahir S., Makary M.S., Longworth S., Lang E.V., Krishna N.G., Mayr N.A., Nguyen X.V. (2020). Communication and Team Interactions to Improve Patient Experiences, Quality of Care, and Throughput in MRI. Top. Magn. Reason. Imaging.

[29] Hashim M.J. (2017). Patient-Centered Communication: Basic Skills. Am. Fam. Physician.

[30] Gaspar R., Domings S., Diniz A.M., Falanga R., Graffigna G. (2016). Barriers to and facilitators of older adult’s adherence to health recommendations: Towards an engaging two-way health communication. Promoting Patient Engagement and Participation for Effective Healthcare Reform.

[31] Chandra S., Mohammadnezhad M., Ward P. (2018). Trust and Communication in a Doctor-Patient Relationship: A Literature Review. J. Healthc. Commun..

[32] Chadderdon A.L., Carns D.R., Pudalov L.R., McKernan L.C., Honce J.M. (2020). Underlying Mechanisms of Psychological Interventions in Magnetic Resonance Imaging and Image-Guided Radiology Procedures. Top. Magn. Reason. Imaging.

[33] Itri J.N. (2015). Patient-centered Radiology. Radiographics.

[34] Dewey M., Schink T., Dewey C.F. (2007). Claustrophobia during magnetic resonance imaging: Cohort study in over 55,000 patients. J. Magn. Reason. Imaging.

[35] Foldes Z., Ala-Ruona E., Burguer B., Orsi G. (2017). Anxiety reduction with music and tempo synchronization on magnetic resonance imaging patients. Psychomusicol. Music Mind Brain.

[36] Lo Re G., De Luca R., Muscarneri F., Dorangricchia P., Picone D., Vernuccio F., Salerno S., La Tona G., Pinto A., Midiri F. (2016). Relationship between anxiety level and radiological investigation. Comparison among different diagnostic imaging exams in a prospective single-center study. Radiol. Med..

[37] Grey S.J., Price G., Mathews A. (2000). Reduction of anxiety during MR imaging: A controlled trial. Magn. Reason. Imaging.

[38] Tugwell J.R., Goulden N., Mullins P. (2018). Alleviating anxiety in patients prior to MRI: A pilot single-centre single-blinded randomised controlled trial to compare video demonstration or telephone conversation with a radiographer versus routine intervention. Radiography.

[39] Jöreskog K.G., Goldberger A.S. (1975). Estimation of a model with multiple indicators and multiple causes of a single latent variable. J. Am. Stat. Assoc..

[40] Sociedade Portuguesa de Ginecologia (2021). Consenso Nacional Sobre Menopausa 2021.

[41] Lovibond P.F., Lovibond S.H. (1995). The structure of negative emotional states: Comparison of the Depression Anxiety Stress Scales (DASS) with the Beck Depression and Anxiety Inventories. Behav. Res. Ther..

[42] Pais-Ribeiro J.L., Honrado A., Leal I. (2004). Contribuição para o estudo da adaptação portuguesa das Escalas de Ansiedade, Depressão e Stress (EADS) de 21 itens de Lovibond e Lovibond. Psicol. Saúde Doenças.

[43] Jöreskog K.G. (2005). Structural Equation Modeling with Ordinal Variables Using LISREL. https://ssicentral.com/wp-content/uploads/2021/04/lis_ordinal.pdf.

[44] Satorra A., Bentler P.M., von Eye A., Clogg C.C. (1994). Corrections to test statistics and standard erros in covariance structure analysis. Latente Variable Analysis.

[45] Hu L.T., Bentler P.M. (1998). Fit indices in covariance structure modeling: Sensitivity to underparameterized model misspecification. Psychol. Methods.

[46] Fornell C., Larcker D.F. (1981). Evaluating Structural Equation Models with Unobservable Variables and Measurement Error. J. Mark. Res..

[47] Nunnally J.C., Bernstein I.H. (1994). Psychometric Theory.

[48] Cohen J. (2002). Statistical Power Analysis for the Behavioral Sciences.

[49] Munn Z., Pearson A., Jordan Z., Murphy F., Pilkington D., Anderson A. (2015). Patient Anxiety and Satisfaction in a Magnetic Resonance Imaging Department: Initial Results from an Action Research Study. J. Med. Imaging Radiat. Sci..

[50] McLean C.P., Anderson E.R. (2009). Brave men and timid women? A review of the gender differences in fear and anxiety. Clin. Psychol. Rev..

[51] Meyers-Levy J., Loken B. (2015). Revisiting gender differences: What we know and what lies ahead. J. Consum. Psychol..

[52] Dewey R.S., Ward C., Junor A., Horobin A. (2021). Talk to us! Communication is a key factor in improving the comfort of MRI research participants. Health Expect..

[53] Stogiannos N. (2019). Reducing patient’s psychological stress. A guide for MR technologists. Hell. J. Radiol..

[54] Lang E.V., Ward C., Laser E. (2010). Effect of team training on patients’ ability to complete MRI examinations. Acad. Radiol..

[55] Protheroe J., Whittle R., Bartlam B., Estacio E.V., Clark L., Kurth J. (2017). Health literacy, associated lifestyle and demographic factors in adult population of an English city: A cross-sectional survey. Health Expect..

[56] Beauchamp A., Buchbinder R., Dodson S., Batterham R.W., Elsworth G.R., McPhee C., Sparkes L., Hawkins M., Osborne R.H. (2015). Distribution of health literacy strengths and weaknesses across socio-demographic groups: A cross-sectional survey using the Health Literacy Questionnaire (HLQ). BMC Public Health.

[57] Tsai T.I., Yu W.R., Lee S.D. (2018). Is health literacy associated with greater medical care trust?. Int. J. Qual. Health Care.

[58] Hyde L.L., JMackenzie L., Boyes A.W., Symonds M., Brown S., Sanson-Fisher R. (2018). Medical Imaging Outpatients’ Experiences With Receiving Information Required for Informed Consent and Preparation: A Cross-Sectional Study. J. Patient Exp..

[59] Wagenmakers E.J., Marsman M., Jamil T., Ly A., Verhagen J., Love J., Selker R., Gronau Q.F., Šmíra M., Epskamp S. (2018). Bayesian inference for psychology. Part I: Theoretical advantages and practical ramifications. Psychon. Bull. Rev..

[60] Wagenmakers E.J., Love J., Marsman M., Jamil T., Ly A., Verhagen J., Selker R., Gronau Q.F., Dropmann D., Boutin B. (2018). Bayesian inference for psychology. Part II: Example applications with JASP. Psychon. Bull. Rev..

[61] (2017). Bayes Like A Baws: Interpreting Bayesian Repeated Measures [Webpage on the Internet]. https://www.cogsci.nl/blog/interpreting-bayesian-repeated-measures-in-jasp.

[62] Asante S., Acheampong F. (2021). Patients’ knowledge, perception, and experience during magnetic resonance imaging in Ghana: A single centre study. Radiography.

[63] Lubetkin E.I., Zabor E.C., Isaac K., Brennessel D., Kemeny M.M., Hay J.L. (2015). Health literacy, information seeking, and trust in information in Haitians. Am. J. Health Behav..

[64] Madl J., Janka R., Bay S., Rohleder N. (2022). MRI as a Stressor: The Psychological and Physiological Response of Patients to MRI, Influencing Factors, and Consequences. J. Am. Coll. Radiol..

[65] Borem L.M.A., Figueiredo M.F.S., Silveira M.F., Neto J.F.R. (2013). The knowledge about diagnostic imaging methods among primary care and medical emergency physicians. Radiol. Bras..

[66] Tugwell-Allsup J., Pritchard A.W. (2018). The experience of patients participating in a small randomised control trial that explored two different interventions to reduce anxiety prior to an MRI scan. Radiography.

[67] Carlsson S., Carlsson E. (2013). ‘The situation and the uncertainty about the coming result scared me but interaction with the radiographers helped me through’: A qualitative study on patients’ experiences of magnetic resonance imaging examinations. J. Clin. Nurs..

[68] Graffigna G. (2016). Promoting Patient Engagement and Participation for Effective Healthcare Reform.

[69] Head M.L., Holman L., Lanfear R., Kahn A.T., Jennions M.D. (2015). The extent and consequences of p-hacking in science. PLoS Biol..

[70] Shadish W.R., Cook T.D., Campbell D.T. (2002). Designs for Generalized Causal Inference.

[71] Paulhus D.L., Zeigler-Hill V., Schackelford T.K. (2017). Socially desirable responding on self-reports. Encyclopedia of Personality and Individual Differences.

[72] Paunonen S.V., LeBel E.P. (2012). Socially desirable responding and its elusive effects on the validity of personality assessments. J. Pers. Soc. Psychol..

[73] Diniz A.M., Amado N. (2014). Procedures for successful data collection through psychological tests in the elderly. Psicol. Reflexão Crítica.

[74] Morrow M., Waters J., Marris E. (2011). MRI for breast cancer screening, diagnosis, and treatment. Lancet.

[75] Oztek M.A., Brunnquell C.L., Hoff M.N., Boulter D.J., Mossa-Basha M., Beauchamp L.H., Haynor D.L., Nguyen X.V. (2020). Practical Considerations for Radiologists in Implementing a Patient-friendly MRI Experience. Top. Magn. Reason. Imaging.

[76] Ferguson C.J. (2009). An effect size primer: A guide for clinicians and researchers. Prof. Psychol. Res. Pract..

[77] Field A. (2018). Discovering Statistics Using IBM SPSS Statistics.

